# Heterologous Matrix Metalloproteinase Gene Promoter Activity Allows *In Vivo* Real-time Imaging of Bleomycin-Induced Lung Fibrosis in Transiently Transgenized Mice

**DOI:** 10.3389/fimmu.2017.00199

**Published:** 2017-03-01

**Authors:** Fabio Franco Stellari, Francesca Ruscitti, Daniela Pompilio, Francesca Ravanetti, Giulia Tebaldi, Francesca Macchi, Andrea Elizabeth Verna, Gino Villetti, Gaetano Donofrio

**Affiliations:** ^1^Chiesi Farmaceutici S.p.A., Corporate Pre-Clinical R&D, Parma, Italy; ^2^Dipartimento di Scienze Medico Veterinarie, Università di Parma, Parma, Italy

**Keywords:** idiopathic pulmonary fibrosis, bleomycin, matrix metalloproteinase-1 promoter, bioluminescence, fluorescent molecular tomography

## Abstract

Idiopathic pulmonary fibrosis is a very common interstitial lung disease derived from chronic inflammatory insults, characterized by massive scar tissue deposition that causes the progressive loss of lung function and subsequent death for respiratory failure. Bleomycin is used as the standard agent to induce experimental pulmonary fibrosis in animal models for the study of its pathogenesis. However, to visualize the establishment of lung fibrosis after treatment, the animal sacrifice is necessary. Thus, the aim of this study was to avoid this limitation by using an innovative approach based on a double bleomycin treatment protocol, along with the *in vivo* images analysis of bleomycin-treated mice. A reporter gene construct, containing the luciferase open reading frame under the matrix metalloproteinase-1 promoter control region, was tested on double bleomycin-treated mice to investigate, in real time, the correlation between bleomycin treatment, inflammation, tissue remodeling and fibrosis. Bioluminescence emitted by the lungs of bleomycin-treated mice, corroborated by fluorescent molecular tomography, successfully allowed real time monitoring of fibrosis establishment. The reporter gene technology experienced in this work could represent an advanced functional approach for real time non-invasive assessment of disease evolution during therapy, in a reliable and translational living animal model.

## Introduction

Idiopathic pulmonary fibrosis (IPF), the most common of the idiopathic interstitial pneumonias, is a devastating condition that carries a prognosis worse than that of many cancers. The median survival of patients with well-defined IPF is less than 5 years after diagnosis (1, 2). Fibrosis, in general, is defined by the excessive accumulation of fibrous connective tissue (components of the extracellular matrix such as collagen and fibronectin) in and around inflamed or damaged tissue, which can lead to permanent scarring, organ malfunction, and, ultimately, death, as is the case for IPF.

A better understanding of the pathogenesis of IPF is critical for the identification of new therapeutic targets as well as molecules that may serve as surrogate markers for clinically significant endpoints. During the last 20 years, the knowledge regarding the pathogenesis of pulmonary fibrosis has substantially improved, and potential targets for novel therapies have been identified. In contrast, despite many potentially effective anti-fibrotic therapeutics have been generated, only few of them accomplished their translation in clinical application. The *in vivo* study, to recapitulate the pathogenesis, the disease phenotype as well as the effect of a drug on the disease outcome, represents the main obstacle to be overcome during the last steps of development of such compounds. Different models have been built over time to examine pulmonary fibrosis. These methods include radiation damage, administration of asbestos and silica, and administration of fibrinogenic cytokines and bleomycin (3). This later method became the standard agent to induce experimental pulmonary fibrosis in animals such as small laboratory animals (including mice, rats, guinea pigs, hamsters, and rabbits) (3, 4) and large animals (including non-human primates, horses, dogs, and ruminants) (3, 5, 6). Bleomycin was initially discovered as chemotherapeutic antibiotic made by the bacterium *Streptomyces verticillus* (7), and it is still used for some forms of neoplasia (8). Its use in animal models of pulmonary fibrosis is based on the fact that fibrosis is a side effect of this agent in human cancer therapy (8).

As in all the models focused on modeling a chronic pulmonary fibrotic state in animals, fibrotic lesions occur 2–3 weeks after bleomycin injury. The time needed to develop these lesions coupled with the necessity to investigate drug effects after interventional treatment on established fibrosis to dissect the anti-fibrotic effects from anti-inflammatory and radical scavenging activities make the bleomycin model a very time consuming one. Moreover, for the detection and quantification of bleomycin-induced lung damage/injury most of the analysis routinely performed, including biochemical measures of matrix deposition and morphometrical and histological analysis, were based on animal sacrifice for postmortem analysis, thus preventing the reuse of the animal for further investigation.

Although other imaging technologies have been used to follow the establishment of lung fibrosis in real time, such as micro-CT, PET, MRI, and cathepsin-activated fluorescent probes (9–11), we propose an innovative and easy *in vivo* bioluminescent imaging (BLI) approach for drug discovery purpose.

## Materials and Methods

### Reagents

*In vivo* JetPEI DNA transfection reagent (Polyplus Transfection) was obtained from Euroclone (Milan, Italy), and d-luciferin was obtained from Perkin Elmer Inc. (Boston, MA, USA). Bleomycin sulfate from *S. verticillus* (Sigma B2434).

### Experimental Animals

Female inbred C57Bl/6 (7- to 8-week old) mice were purchased from Harlan Laboratories Italy (San Pietro al Natisone, Udine, Italy). Prior to use, animals were acclimatized for at least 5 days to the local vivarium conditions (room temperature: 20–24°C; relative humidity: 40–70%; 12-h light–dark cycle), having free access to standard rodent chow and softened tap water. All animal experiments described herein were approved by the intramural animal-welfare committee for animal experimentation of Chiesi Farmaceutici and comply with the European Directive 2010/63 UE, Italian D.Lgs 26/2014 and the revised “Guide for the Care and Use of Laboratory Animals.”

### *In Vivo* Gene Delivery

The pMMP-1-Luc was obtained by subcloning a 1101 *Mlu*I/*Xho*I bMMP-1 promoter sequence, digested with the same enzymes from pEX-A2 (Eurofins) and inserted in Mlu/*Xho*I digested pGL3 basic vector (Promega). We applied *in vivo* JetPEI (Polyplus Transfection) as a carrier for delivering DNA to lung tissues. The DNA and JetPEI mix was formulated according to the product manual with a final N/P ratio of 7. Briefly, 50 μg of bMMP-1-luc and 7 μl of JetPEI were both diluted into 200 μl 5% glucose. The two solutions were then mixed and incubated for 15 min at room temperature. The entire mixture was injected intravenously into C57Bl/6 mice, and the expression of pbMMP-1-Luc was monitored through imaging with an IVIS imaging system.

### *In Vivo* Bioluminescence Imaging

Transfection *per se* causes a mild lung inflammatory response and matrix metalloproteinases (MMPs)-1Luc activation that is detectable by BLI up to 3–4 days after DNA injection and disappears completely after 1 week (12, 13). One week after DNA delivery, the transient transgenic mice were injected intraperitoneally (i.p.) with luciferin (150 mg/kg), and BLI was recorded in order to check the baseline activation of the MMP-1 pathway. Briefly, 10 and 15 min following luciferin injection, mice were lightly anesthetized with isoflurane (2.5% in oxygen), and images were obtained using the IVIS imaging system: a total of photons emitted from the chest of the mice was quantified using Living Image^®^ software (Perkin Elmer Inc., Boston, MA, USA). The following day, mice were intratracheally challenged with bleomycin or with saline, and BLI was recorded at 4, 24, 48 h, and every 7 days for 3 weeks. Data were expressed as mean folds of induction (FOI) over the baseline activation of each mouse (12, 14, 15).

### *In Vivo* Fluorescent Molecular Tomography (FMT) Imaging

Bleomycin-treated and control mice were injected intravenously with MMPSense680 (Perkin Elmer Inc., Boston, MA, USA) at 80 nmol/kg at 7, 14, and 21 days and imaged 24 h after probe injection. MMPSense680 is a metalloproteinase activatable fluorescent *in vivo* imaging agent that is activated by key MMPs including MMP-2, -3, -9, and -13. MMPSense 680 is optically silent in its inactivated state and becomes highly fluorescent following metalloprotease-mediated activation (13, 16).

Mice were anesthetized using isoflurane (2.5% in oxygen) and depilated to minimize fur interference with fluorescent signal. Depilatory cream was applied thickly on skin over the upper torso (front, back, and sides) of each mouse, rinsed off with warm water, and reapplied until all fur had been removed.

Bleomycin-challenged and control mice were then imaged using the FMT 2500 *in vivo* imaging system (Perkin Elmer Inc., Boston, MA, USA). Briefly, the anesthetized mice were carefully positioned in the imaging cassette, which was then placed into the FMT imaging chamber. A near infrared (NIR) laser diode transilluminated (i.e., passed light through the body of the animal to be collected on the opposite side) the thorax region, with signal detection occurring *via* a thermoelectrically cooled charge-coupled device camera placed on the opposite side of the imaged animal. Appropriate optical filters allowed collection of both fluorescence and excitation data sets, and the multiple source-detector fluorescence projections were normalized to the paired collection of laser excitation data. The entire image acquisition sequence took approximately 4–5 min per mouse.

The collected fluorescence data were reconstructed by FMT 2500 system with TrueQuant software version 2.2 (Perkin Elmer Inc., Boston, MA, USA) for the quantification of three-dimensional fluorescence signal within the lungs. The total amount of lung fluorescence (in picomoles) was automatically calculated from internal standards generated with known concentrations of appropriate NIR dyes.

### Bleomycin Administration

For intratracheal administrations, 50 μl/mouse either saline or bleomycin (1 mg/kg) were given directly through the tracheal cannula instillation under isoflurane (2.5% in oxygen) anesthesia, using a small laryngoscope (Penn-Century Inc., Philadelphia, PA, USA) to visualize the trachea. In brief, mice were anesthetized and challenged intratracheally with saline, or with bleomycin single dose at day 0 or bleomycin double treatment at day 0 and day 4.

### Bronchoalveolar Lavage, Cytokines, and MMPs

At days 7, 14, and 21 days posttreatment the first bleomycin challenge, animals were anesthetized with isoflurane and sacrificed by bleeding from the abdominal aorta. Bronchoalveolar lavage fluid (BALF) was collected by gently washing the lungs with 0.6 mL sterile solution [Hank’s balanced salt solution × 10; ethylenediaminetetraacetic acid 100 mM; 4-(2-hydroxy-ethyl)-1-piperazineethansulphonic acid 1 mM; and distilled water] for three times in the bronchial tree and collected for subsequent analysis (14). After centrifugation at 400 *g* for 10 min, the BAL supernatants were frozen at −80°C for simultaneous quantitation of multiple cytokines/chemokines using a Bio-Plex™ Cytokine Assay Kit (Bio-Rad Laboratories, Segrate, Milano, Italy). The cell pellet was resuspended in 0.2 ml of PBS. Cell number was counted with an automated cell counter (Dasit XT 1800J). The concentration of MMP proteins in BAL fluid was determined by enzyme-linked immunosorbent assay (ELISA). MMP-2, MMP-9, and TIMP-1 were measured with specific ELISA kit (R&D System), and MMP-1 was measured with Mouse Matrix Metalloproteinase 1 ELISA Kit (MyBioSource) according to the manufacturer’s instructions.

### Histology

Lungs were removed and inflated with a cannula through the trachea by gentle infusion with 0.6 ml of 10% neutral-buffered formalin. After the lungs were placed in a vial containing 10% formalin and fixed for at least 24 h. For the histology, the whole lungs were dehydrated in graded ethanol series, clarified in xylene and paraffin embedded. Sections of 5 μm thick were cut with a rotary microtome (Reichert-Jung 1150/Autocut) in ventral dorsal plane. Fibrotic lesions were assessed morphologically and semiquantitatively graded according to the scale defined by Ashcroft and modified by Hübner et al. (17). Three sections for each lung sample were stained with Masson’s Trichrome, and tissue damage was assessed by a system of 0–8 score. For each section, all the tissue was scored as independent microscopic fields at 20×, where the degree of fibrosis was recorded as that occupying more than half of each field. The final score was expressed as a mean of individual scores observed on all microscopic fields and sections at the magnification of 20×.

### Data Analysis

Data were expressed as the mean and SEM of the mean of *n* animals. Statistical analysis was performed using (PRISM Statistical software v 4.0.3). Changes were compared to the vehicle groups using ANOVA followed by Dunnett’s test. *p < 0.05; **p < 0.01 and ^#^*p* < 0.05; ^##^*p* < 0.01 when changes were compared to each group vs every other group using ANOVA followed by Tukey’s multiple comparison test.

## Results and Discussion

To better understand the pathogenesis of lung fibrotic disorders, different models of pulmonary fibrosis have been developed over the years. Most of them mimic some, but never all features of human IPF, especially the progressive and irreversible nature of the condition. Among currently applied models of experimentally induced pulmonary fibrosis, the administration of bleomycin is used most frequently. Moreover, the bleomycin-induced lung fibrosis animal model has emerged as the best characterized to study IPF disease although this murine model has its own strengths and weaknesses. Bleomycin-induced pulmonary fibrosis is often unpredictable, indeed different factors will affect the nature of the fibrotic response, including the route of administration, preparation, dose, strain, and age of mice (18). Thus, the first objective of the present work was to attempt a different bleomycin-induced lung fibrosis protocols by comparing a single and a double dose of bleomycin intratracheal instillation. The second objective of this work is to monitor lung fibrosis through non-invasive BLI technique using the pulmonary fibrosis mouse model established in this study.

Bleomycin was intratracheally administered as a single (at day 0; 1 mg/kg of bleomycin in 50 μl) or as a double dose (at day 0 and day 4; 1 mg/kg of bleomycin in 50 μl). Two groups of mice (*n* = 12) intratracheally instilled with only vehicle at the same time and volume used for the groups of test mice were used as untreated controls. Bleomycin- and vehicle-treated mice were daily visually monitored for adverse reactions potentially induced by the treatment and weekly weighed for the complete duration of the experimental period. The majority of side effects were reported in the bleomycin double-treated mice that showed a body weight reduction associate with 10% of mortality from 9 to 15 days after bleomycin administration. No weight loss was found in the control group (Figure S1 in Supplementary Material).

**Figure 1:**
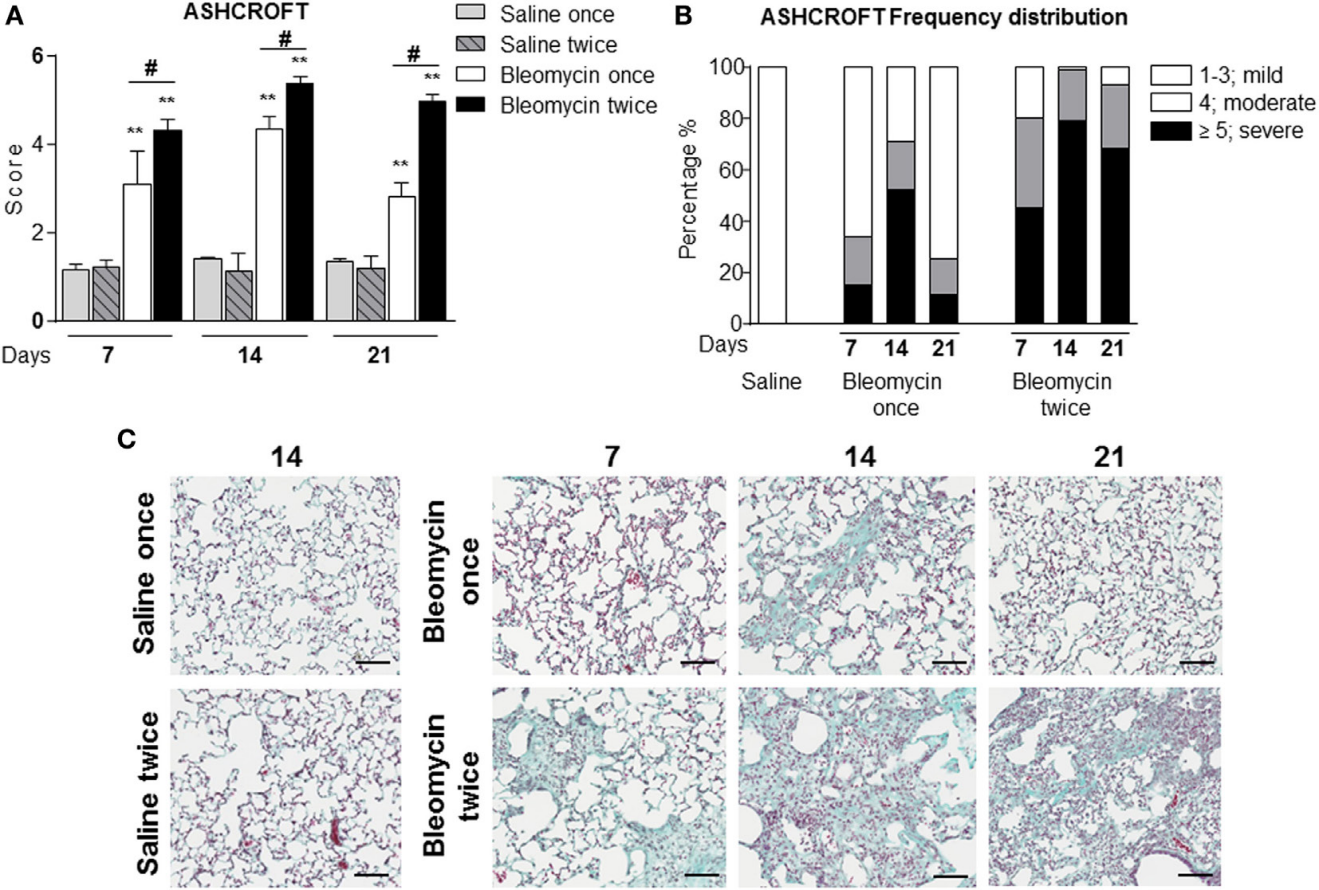
**(A)** Quantification of collagen deposition in the lung of intratracheally instilled with a single (bleomycin once) or double (bleomycin twice) dose of bleomycin and compared with single (saline once) or double (saline twice) dose vehicle (saline) treated mice at 7, 14, and 21 days posttreatment. **(B)** Ashcroft frequency distribution of single (bleomycin once) or double (bleomycin twice) dose of bleomycin and compared with double dose vehicle (saline) treated mice at 7, 14, and 21 days posttreatment. The total Ashcroft score has been divided in three classes (1–3, mild; 4, moderate; ≥5, severe fibrosis). **(C)** Representative images of mouse lung sections treated and untreated with bleomycin and used for the above of the quantification (10×; scale bar is equal to 100 μm). Nine animals were used for each time point, and each time point was repeated three times. The data represent the mean ± SEM of a total of 27 animals. Changes were compared to the vehicle groups using ANOVA followed by Dunnett’s test. *p < 0.05; **p < 0.01 and ^#^*p* < 0.05; ^##^*p* < 0.01 when changes were compared to each group vs every other group using ANOVA followed by Tukey’s multiple comparison test.

For the assessment of fibrosis, a semiquantitative histological analysis was performed based on the scoring system by Ashcroft. Single intratracheal administration revealed high variability intra-experiment; the fibrosis lesions detected by histological analysis were not very severe (Figures 1A–C) and increasing the dose of single bleomycin instillation resulted only in an increased toxicity and mortality (data not shown).

In the single bleomycin-treated group, moderate structural changes were observed at 14 days and tend to resolve spontaneously up to 3 weeks (Figures 1A,B). Histology pictures showed the formation of only single fibrotic masses (Figure 1C). The tendency of spontaneous resolution of the lung fibrosis in single treated group represents a real limitation for pharmacological intervention due to restrict therapeutic window allowing only preventing treatment that does not mimic the clinical practice. On the contrary, in the double bleomycin-treated group, histology analysis revealed more severe pattern of fibrosis started from day 7, mainly as single fibrotic masses, and evolved at days 14 as confluent conglomerates of substitutive collagen and remain stable till day 21 (Figures 1A–C).

**Figure 2:**
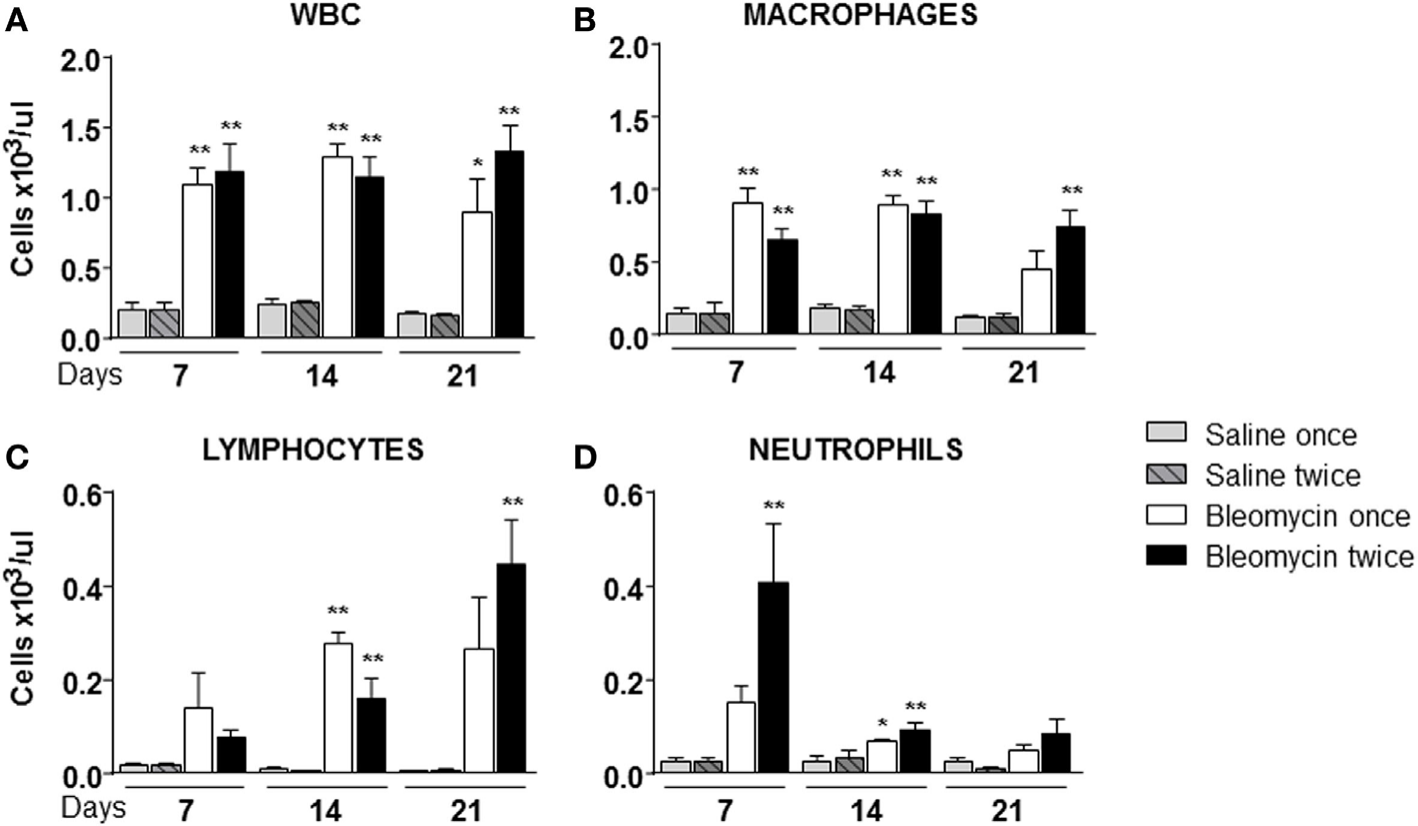
**Cellular infiltration into the bronchoalveolar lavage fluid (BALF) of mice lung intratracheally instilled with a single (bleomycin once) or double (bleomycin twice) dose of bleomycin and compared with single (bleomycin once) or double (bleomycin twice) dose vehicle (saline) treated control groups**. Cellular infiltration was detected at 7, 14, and 21 days posttreatment. The amount of white blood cells (WBC) **(A)**, macrophages **(B)**, lymphocytes **(C)**, neutrophils **(D)**, and monocytes found in BALF was expressed as number of cells per microliter. Nine animals were used for each time point, and each time point was repeated three times. The data represent the mean ± SEM of a total of 27 animals. Changes were compared to the vehicle groups using ANOVA followed by Dunnett’s test. *p < 0.05; **p < 0.01 and ^#^*p* < 0.05; ^##^*p* < 0.01 when changes were compared to each group vs every other group using ANOVA followed by Tukey’s multiple comparison test.

Starting from the assumption that bleomycin is a lung pro-inflammatory molecule and white blood cells (WBC) should be recruited in an inflamed tissue, WBC content in the BALF of bleomycin-treated mice was compared with those treated with vehicle. As expected WBC in BALF of bleomycin-treated mice, as monitored at days 7, 14, and 21, was strongly higher (1.09 ± 0.12 × 10^3^ cell/μl for single treatment and 1.18 ± 0.19 × 10^3^ cell/μl or double treatment) than in the vehicle treated groups (0.2 ± 0.05 × 10^3^ cell/μl), but no differences between single and double treatment were observed (Figure 2A). Interestingly, lymphocyte fraction in the double-treated group increased at day 21 (Figure 2C); macrophage fraction increased at each time of treatment and remained high mainly in double-treated group (Figure 2B); in contrast, no significant increase of the neutrophil fraction was observed in single bleomycin-treated mice. Whereas for bleomycin double treated mice, a significant increase at day 7 was observed (Figure 2D). Therefore, double administration of bleomycin increase, in general, WBC infiltration into the lung.

**Figure 3:**
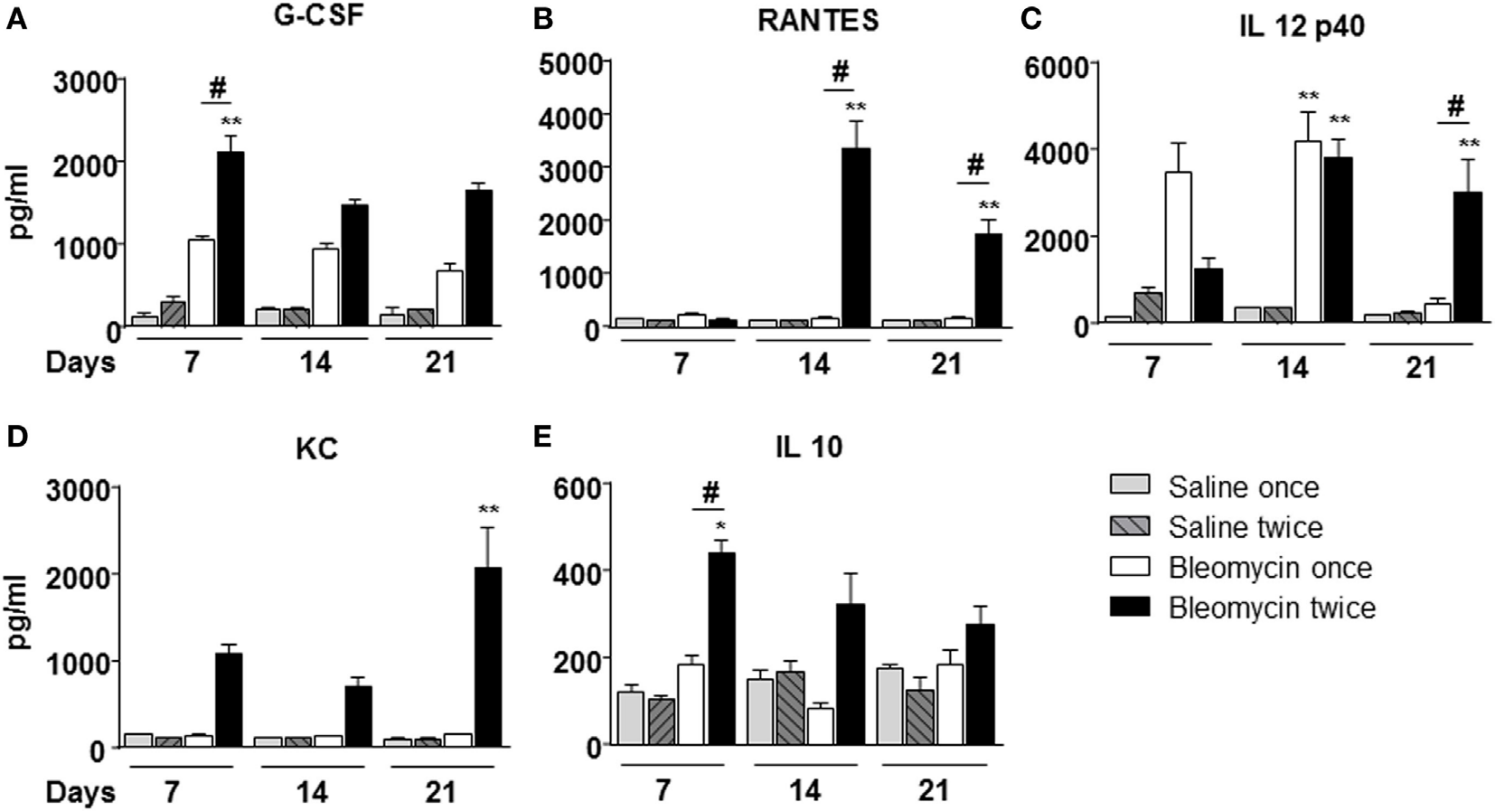
**Cytokines/chemokines (A) G-CSF; (B) RANTES; (C) IL 12p40; (D) KC; (E) IL 10, determination into the bronchoalveolar lavage fluid of mice lung intratracheally instilled with a single (bleomycin once) or double (bleomycin twice) dose of bleomycin and compared with single (bleomycin once) or double (bleomycin twice) dose of vehicle (saline) treated control groups**. Determination was made at 7, 14, and 21 days posttreatment. Nine animals were used for each time point, and each time point was repeated three times. The data represent the mean ± SEM of a total of 27 animals. Changes were compared to the vehicle groups using ANOVA followed by Dunnett’s test. *p < 0.05; **p < 0.01 and ^#^*p* < 0.05; ^##^*p* < 0.01 when changes were compared to each group vs every other group using ANOVA followed by Tukey’s multiple comparison test.

**Figure 4:**
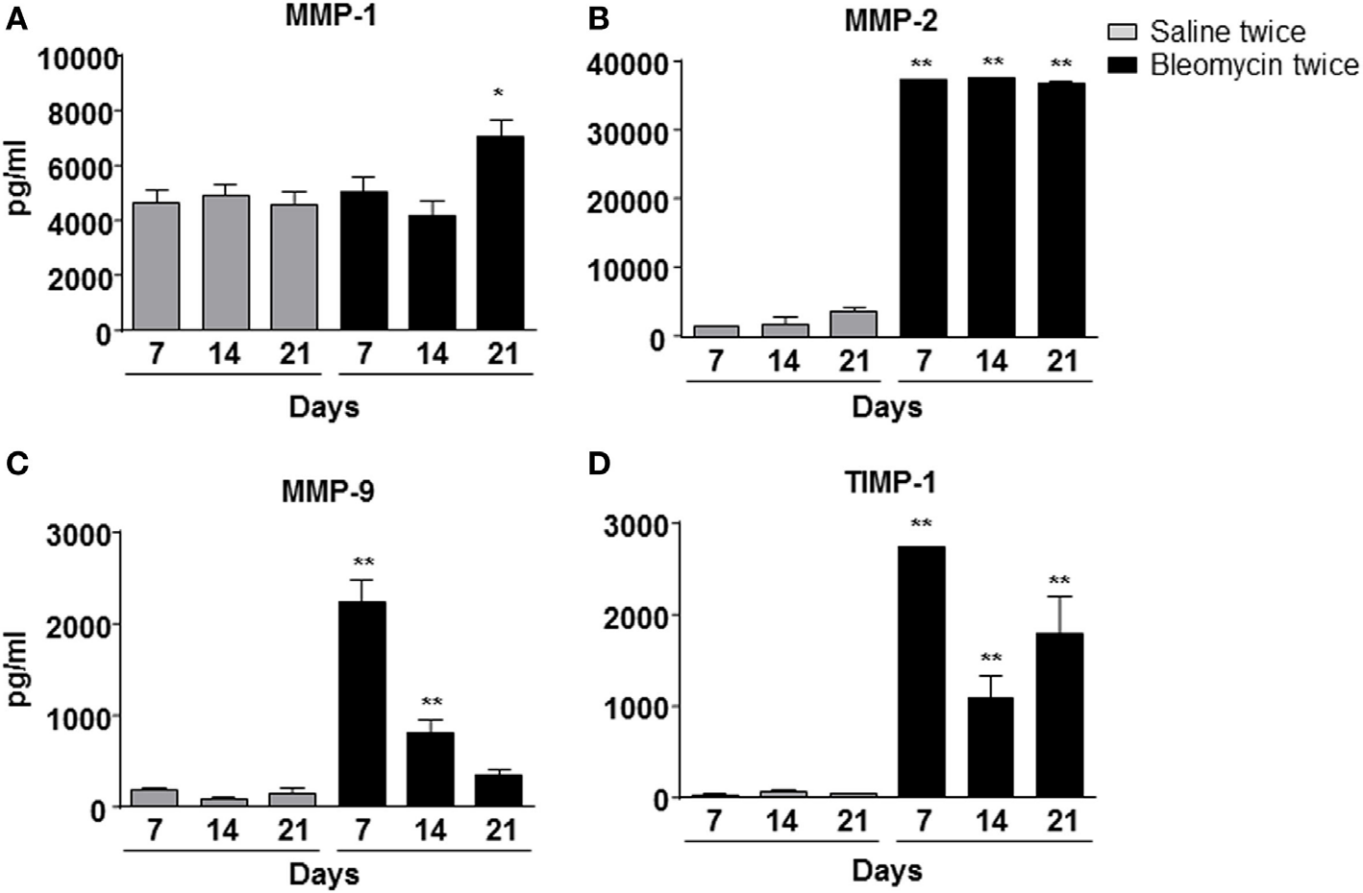
**Matrix metalloproteinase-1 (MMP-1) (A), MMP-2 (B), MMP-9 (C), and TIMP-1 (D) expression into the mice lung bronchoalveolar lavage fluid, intratracheally instilled with double (bleomycin twice) dose of bleomycin and compared with vehicle (saline) treated control mice groups**. MMP-1, MMP-2, MMP-9, and TIMP-1 expression was detected at 7, 14, and 21 days posttreatment. Nine animals were used for each time point, and each time point was repeated three times. The data represent the mean ± SEM of a total of 27 animals. Changes were compared to the vehicle groups using ANOVA followed by Dunnett’s test. *p < 0.05; **p < 0.01.

To correlate WBC infiltration and inflammation, a set of cytokines and chemokines [chemokine (C–X–C motif) ligand 1 (CXCL1 also KC), granulocyte colony-stimulating factor, interleukin 10, chemokine (C–C motif) ligand 5 (CCL5 also RANTES), and interleukin-12 subunit p40] were estimated by cytokine multiplex assay in the BALF. Indeed, all cytokines/chemokines estimated significantly increased in bleomycin double-treated mice with respect to the single bleomycin-treated mice and the untreated controls (Figure 3). The inflammatory response to double bleomycin treatment was further corroborated by the presence of MMP-1 (Figure 4A), MMP-2 (Figure 4B), MMP-9 (Figure 4C), and their inhibitor, TIMP-1 proteins (Figure 4D) by ELISA in the BALFs. All the data described above were satisfactorily in agreement with the increase of collagen deposition and maintenance in double treatment with bleomycin. The correlation between bleomycin double treatment, WBC infiltration, cytokine expression, MMP expression, and collagen deposition prompted us on the potential use of MMP gene as a marker for an *in vivo* real time monitoring of bleomycin-induced lung fibrosis. After an accurate comparison between single vs double bleomycin administration, we decided to use only the double protocol instillation for further experiments.

For translating the experimental setting to a different animal species different than mouse, it was decided to use heterologous DNA sequences coming from the bovine MMP-1 gene (“Bos_taurus.UMD3.1.68” reference genome). A reporter gene construct containing a 1,089 bp minimal MMP-1 promoter sequence including the putative TATAbox and most of the 5′-UTR was cloned in front of the firefly luciferase gene contained in the pGL3 basic vector to generate pbMMP-1-Luc (19).

The possibility to monitor longitudinally in non-invasive way, the activation of MMP-1 in the same mice is step a forward in investigating the molecular mechanism linked to fibrosis progression, with the advantage of reducing variability intra-experiments and the number of animals required.

**Figure 5:**
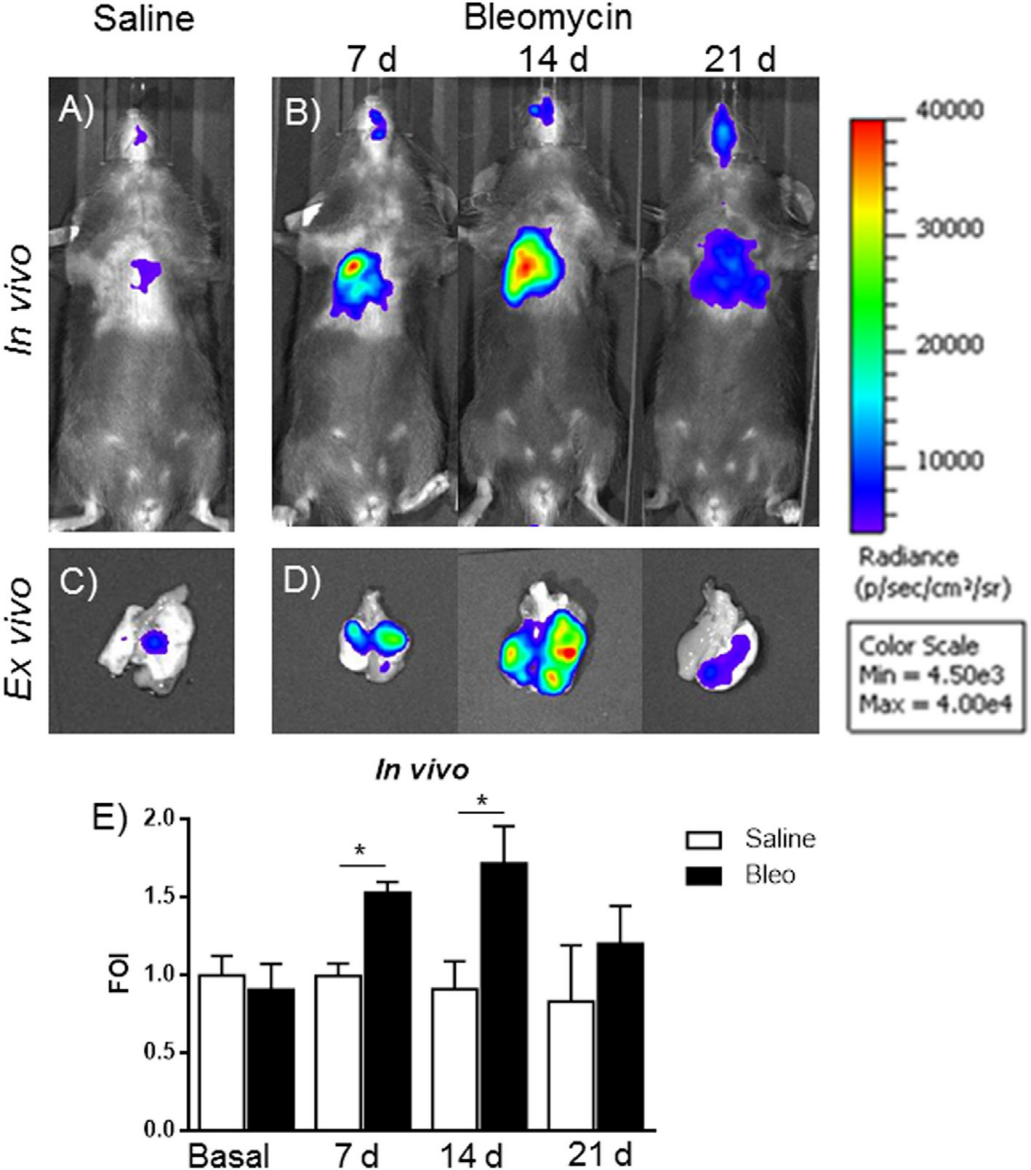
***In vivo* (A,B) and *ex vivo* (C,D) representative images of pbMMP-1-Luc transiently transgenized mice treated with saline (A,C) or bleomycin (B,D) at different time posttreatment (7, 14, and 21 days posttreatment)**. Mice were monitored before the treatment to get the baseline and at 4, 24, and 48 h posttreatment by *in vivo* image analysis drawing a region of interest over the chest and using an IVIS imaging system (Perkin Elmer Inc., Boston, MA, USA). Light emitted was acquired from specific regions by Living Image^®^ software (Perkin Elmer Inc., Boston, MA, USA) as photon per second per square centimeter and normalized as folds of induction respect to the saline-treated control **(E)**. Statistical differences were tested by one-way ANOVA followed by Dunnett’s *post hoc* test for group comparisons. Nine animals were used for each time point, and each time point was repeated three times. The data represent the mean ± SEM of a total of 27 animals. Changes were compared to the vehicle groups using ANOVA followed by Dunnett’s test. *p < 0.05; **p < 0.01.

The gene delivery technology has extensively been used in our lab, and we know that the transient transgene expression could last for more the 60 days (14), starting from this assumption, mice were transiently transgenized with pbMMP-1-Luc. The DNA, when is systemically administered, is able to transfect the 50% of epithelial and endothelial cells in the lung (13). One week post-transgenization, all the mice were first imaged to detect a residual BLI signal (12, 13), and, at the same time, they were intratracheally instilled with bleomycin. At day 4, bleomycin was given for the second time to all the mice. At different time points (7, 14, and 21 days) after bleomycin treatment, mice were monitored for BLI expression and MMPS activation in the lung by IVIS and FMT *in vivo* imaging. *In vivo* and *ex vivo* BLI images (Figures 5A–D) showed that mice responded to bleomycin insult with a maximal signal at 14 days. In contrast, no signal was observed for control mice treated with saline or those transiently transgenized with the empty vector (data not shown). The difference in term of time between MMP-1 detected into the BALF and MMP-1 promoter activation measured by BLI could be explained by the fact that MMP transcription takes place early respect to lung tissue chronic inflammation and tissue modification, which need longer time.

Signal intensity was then expressed as FOI where the saline-treated control was considered as 1 (Figure 5E). A further control was established with mice transiently transgenized with STAT-1-Luc vector because STAT-1 is not controlled by transcription but by its activation through phosphorylation upon the interaction with JAK, in fact no signal was detected after bleomycin treatment (Figure S2 in Supplementary Material).

**Figure 6:**
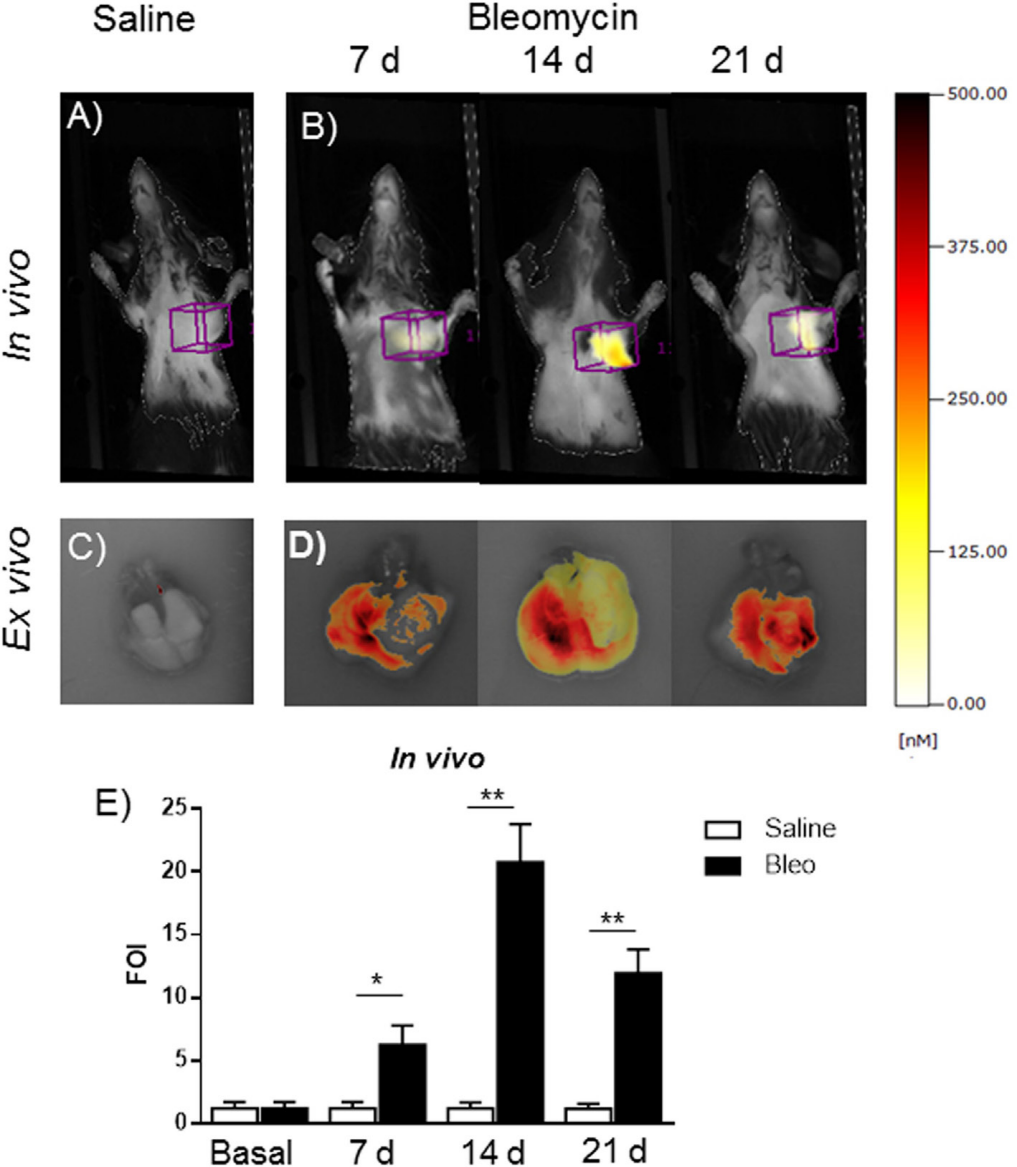
***In vivo* (A,B) and *ex vivo* (C,D) fluorescent molecular tomography (FMT) representative images of mice injected with MMPSense 680 treated with saline or double treated with bleomycin at different time posttreatment (7, 14, and 21 days)**. The total amount of lung fluorescence signal was automatically calculated by FMT with True Quant software version 2.2 (Perkin Elmer Inc., Boston, MA, USA). **(E)** Nine animals were used for each time point, and each time point was repeated three times. The data represent the mean ± SEM of a total of 27 animals. Changes were compared to the vehicle groups using ANOVA followed by Dunnett’s test. *p < 0.05; **p < 0.01.

This *in vivo* bioluminescent analysis demonstrated the specificity of the system even using a promoter coming from a different animal species. The role of MMPs in the present bleomycin double treatment protocol was further corroborated by using the FMT imaging technology, injecting the mice with MMPSense 680 at different time points (7, 14, and 21 days). In fact, bleomycin-treated mice were found to be responsive in terms of lung MMP protein expression as revealed by the activation of MMP-specific fluorescent probes (Figure 6). *In vivo* and *ex vivo* time course results were reported as mean ± SEM and significance attributed when *p < 0.05 or **p < 0.01. These results underlines the possible use of two molecular readouts in the same animal *in vivo*, which represents a step ahead toward the understanding of a very complex disease such as IPF and other diseases involving MMP activation and collagen deposition.

Herein, attempting to correlate bleomycin-induced lung inflammation, tissue damage and remodeling processes, several MMPs and their inhibitor, such as TIMP-1, in double bleomycin-treated mice were found expressed. Although MMP-2 and MMP-9 were better upregulated respect to MMP-1 in our experimental setting, it was of greater significance focusing on MMP-1 for several reasons. The molecular events involving IPF in lethal lung disorders, such us pulmonary fibrosis and including bleomycin-induced fibrosis, have not been completely elucidated; fibrinogenic pathway activation, along with the recruitments of epithelial cells to epithelial–mesenchymal transition phenomena, seem to have a pivotal role, and MMP-1 is one of the most expressed gene (20–23). MMP-1 is an archetypical collagenase, whose substrates were represented by collagenase type I, II, and/or III. Further, several investigations have demonstrated that MMP-1 plays an activating role in detaching membrane-linked form of several precursor molecules from the cell surface. After MMP-1 cleavage, the biologically active forms of these molecules can freely diffuse and act into the tissue interstitial space or be transported by the blood or lymphatic stream to distant different places from where they have been produced. Among these precursor molecules, pro-TGF-α, EGF-like ligands, and TGF-β precursor can be listed. MMP-1 has also been proven to process different important mediators, such as pro-TNF-α, pro-IL-1β, L-selectin, α1-antitripsin inhibitor, connective tissue growth factor, complement C1q fraction, IL-8, and growth factor-binding proteins 1 and 3 (24–27). Regarding IL-8, it is important to note that the MMP-cleaved form of this cytokine is 10-fold more potent than the original molecule (28). Indeed, the overexpression of MMP-1 was detected in lung of cigarettes smoking people, people with lung emphysema, with IPF and cancer (29, 30). An important feature for MMP-1 pathway investigation is the identification of a suitable laboratory animal model where MMP-1 gene expression can be easily detected, following a specific stimulus, and manipulated. Human MMP-1 gene and protein are highly conserved among different animal species in terms of structure and function, in fact human MMP-1 has more than 99% of identity with *Bos taurus* and *Capra hircus* (31, 32). Recently, two genes with similar characteristics of human and bovine MMP-1, Mmp1a and Mmp1b, have been identified in mice. These two genes have 82% of similarity while Mmp1a has 58% of similarity with human MMP-1, as well as a similar chromosomal location and surrounding genes, such as MMP-3 and MMP-10, as revealed by chromosome mapping (31–33),. For these reasons and for its collagenase activity, absent in Mmp1b, Mmp1a was considered the natural mouse ortholog of human and bovine MMP-1. Taking advantage from the data previously obtained from the use of a bovine IL-8 luciferase reporter construct, which was successfully employed to transiently transgenize mice, even though mice do not bear a functional IL-8 gene (12, 14, 15), a new reporter construct was generated. Moreover, in different experimental settings using human regulatory sequences for *in vivo* gene delivery in rodents, for reasons we still do not understand, we noticed that bovine sequences seem to be better recognized by the mouse transcriptional apparatus than the human one. For this reason, it was decided to use the bovine MMP-1 promoter than the human ones, and this model could be of great value for drug discovery in human lung diseases.

The proximal promoter of the bovine MMP-1 gene was used to drive the luciferase reporter gene transcription, transiently transgenized mice were generated, intratracheally double treated with bleomycin and luminescence signal intensity monitored at different time points. By this experimental setting, mouse cell signaling and transcriptional apparatus were able to recognize the bovine MMP-1 promoter following the specific fibrotic insult induced by bleomycin treatment. This has allowed us to develop a more reproducible and robust murine model for lung fibrosis based on a double intratracheal administration of bleomycin, and also opened up a new door for *in vivo* monitoring in real time by BLI a specific molecular mechanism linked to pulmonary fibrosis.

The simple system applied for the generation of transient transgenic mice resulted to be a valid alternative to the normal transgenesis. The utility of reporter gene technology makes possible to analyze specific cellular and biological processes in a living animal through *in vivo* imaging methods. BLI from luciferase gene expression, as is the case of the reporter construct employed during this study, is the most widely used. Because mammalian tissues do not naturally emit bioluminescence, *in vivo* BLI has considerable appeal because images can be generated with absence of background signal. BLI requires an expression cassette consisting of the bioluminescence reporter gene (in this case was luciferase) under the control of a selected promoter (in this experimental setting the *B. taurus* gene promoter) driving the reporter. In order to induce light production, the substrate luciferin must be provided by intravascular or intraperitoneal injection. To date, there have been no reports of toxicity related to repeated dosing of substrates (34), and bMMP-1-luciferase transiently transgenized mice could be reutilized.

Thus, the capability to monitor a biological process longitudinally in the same mouse represents an obvious advance for functional as well as pharmacological studies. Although FMT detected a better signal respect to BLI, BLI is more commonly used, versatile, inexpensive, and handy for either drug discovery or basic science. The major drawback for FMT is the dependencies from the commercial probes, which can be very expensive, require continue administration, and need high-skill operators to be reproducible. BLI, through specific promoters driving a reporter gene, is targeting the transcriptional control of a specific molecule, whereas FMT is targeting the activation of proteins, in this specific case the proteases already present in the diseased tissue. Therefore, the two technologies are not completely overlapping, and in our case, the use of FMT was to further validate BLI.

The correlation between bleomycin treatment, lung inflammation, MMP upregulation and collagen deposition is in accordance with the recognized MMP role as a key factor in orchestrating the onset of lung fibrosis, thus further validating the model presented by this work, which is suitable to functional and pharmacological study in a convenient small size experimental animal model aimed to combat a devastating disease such as pulmonary fibrosis and other diseases related to it.

## Ethics Statement

Animal experiments were conducted in compliance with national (Decreto Legislativo numero 26, 4 Marzo 2014) and international laws and policies (Guide for the Care and Use of Laboratory Animals). Animal studies were approved by the Institutional Animal Care and Use Committee at Chiesi Farmaceutici, Parma, Italy.

## Author Contributions

FS and GD conceived the experiments. FS, FRuscitti, DP, FRavanetti, GT, FM, AV, GV, and GD performed the experiments. GD and FS analyzed the data. GD wrote the paper.

## Conflict of Interest Statement

The authors declare that the research was conducted in the absence of any commercial or financial relationships that could be construed as a potential conflict of interest.

## Supplementary Material

Figure S1 **Effect of intratracheally administration of bleomycin on mice body weight**. Nine animals were used for each time point, and each time point was repeated three times. The data represent the mean ± SEM of a total of 27 animals. Changes were compared to the vehicle group using ANOVA followed by Dunnett’s test. **p* < 0.05; ***p* < 0.01.

Figure S2 ***In vivo* (A,B) representative images of STAT-1-Luc transiently transgenized mice treated with saline (A) or bleomycin (B) at different time posttreatment (7, 14, and 21 days posttreatment)**. Mice were monitored before the treatment to get the baseline and at 4, 24, and 48 h posttreatment by *in vivo* image analysis drawing a region of interest over the chest and using an IVIS imaging system (Perkin Elmer Inc., Boston, MA, USA). Light emitted was acquired from specific regions by Living Image^®^ software (Perkin Elmer Inc., Boston, MA, USA) as photon per second per square centimeter and normalized as FOI respect to the saline treated control. **(C)** Statistical differences were tested by one-way ANOVA followed by Dunnett’s *post hoc* test for group comparisons. Each point represents the mean ± SEM of three animals. Changes were compared to the vehicle groups using ANOVA followed by Dunnett’s test. **p* < 0.05; ***p* < 0.01.

## Abbreviations

IPF, idiopathic pulmonary fibrosis; MMP-1, matrix metalloproteinase-1; ELISA, enzyme-linked immunosorbent assay; ORF, open reading frame; FMT, fluorescent molecular tomography; BLI, bioluminescence imaging; BALF, bronchoalveolar lavage fluid; HBSS, Hank’s balanced salt solution; EDTA, ethylenediaminetetraacetic acid; HEPES, 4-(2-hydroxy-ethyl)-1-piperazineethansulphonic acid; FBS, fetal bovine serum.
