# Supplementary figures and images for: Heterologous Matrix Metalloproteinase Gene Promoter Activity Allows In Vivo Real-time Imaging of Bleomycin-Induced Lung Fibrosis in Transiently Transgenized Mice
Source: Front Immunol. 2017 Mar 1;8:199. doi: 10.3389/fimmu.2017.00199 (PMC5331072; doi:10.3389/fimmu.2017.00199)

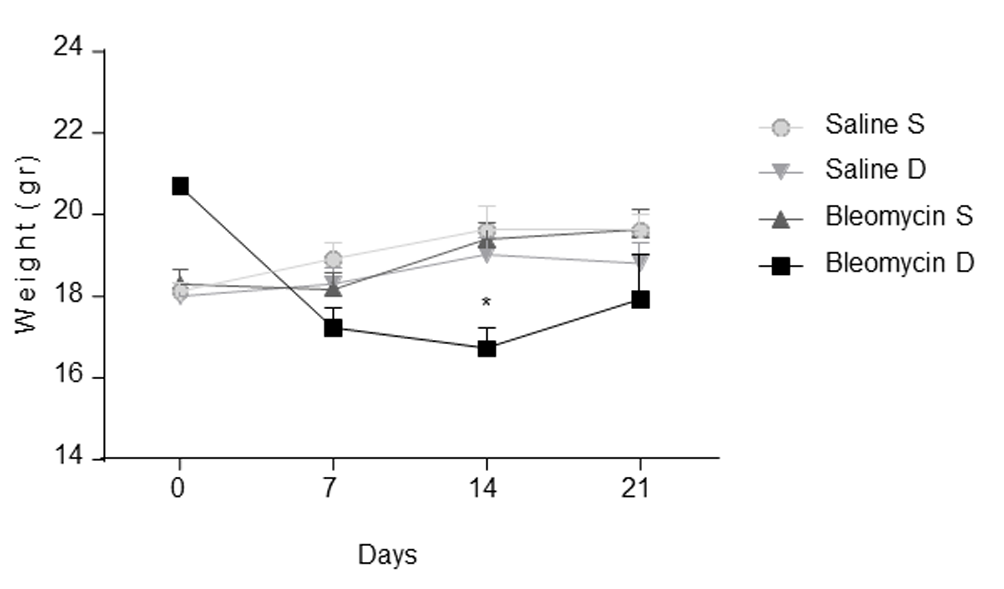

Supplement: Figure S1 — Effect of intratracheally administration of bleomycin on mice body weight. Nine animals were used for each time point, and each time point was repeated three times. The data represent the mean ± SEM of a total of 27 animals. Changes were compared to the vehicle group using ANOVA followed by Dunnett’s test. *p < 0.05; **p < 0.01. [file Image_1.tif]

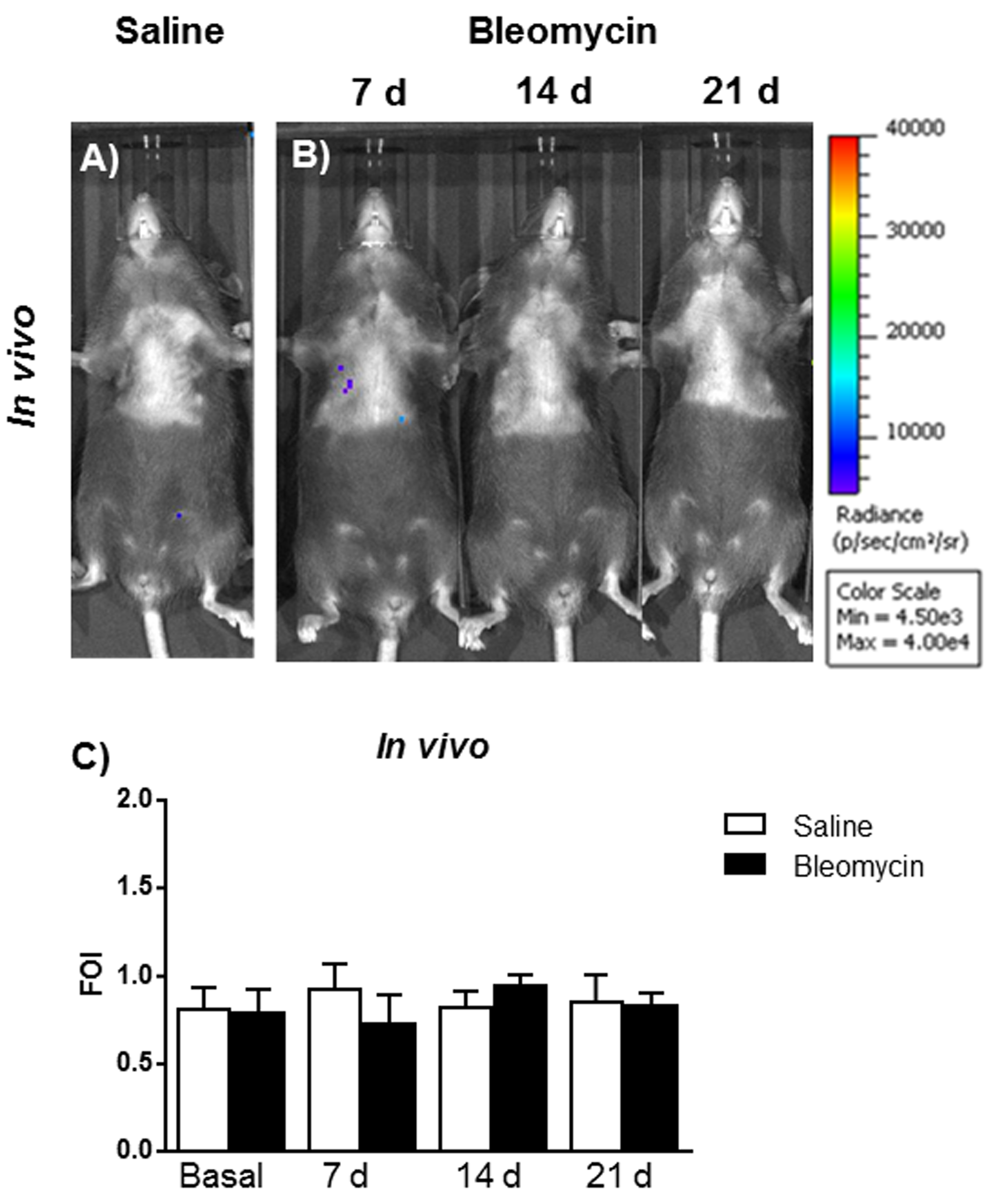

Supplement: Figure S2 — In vivo (A,B) representative images of STAT-1-Luc transiently transgenized mice treated with saline (A) or bleomycin (B) at different time posttreatment (7, 14, and 21 days posttreatment). Mice were monitored before the treatment to get the baseline and at 4, 24, and 48 h posttreatment by in vivo image analysis drawing a region of interest over the chest and using an IVIS imaging system (Perkin Elmer Inc., Boston, MA, USA). Light emitted was acquired from specific regions by Living Image® software (Perkin Elmer Inc., Boston, MA, USA) as photon per second per square centimeter and normalized as FOI respect to the saline treated control. (C) Statistical differences were tested by one-way ANOVA followed by Dunnett’s post hoc test for group comparisons. Each point represents the mean ± SEM of three animals. Changes were compared to the vehicle groups using ANOVA followed by Dunnett’s test. *p < 0.05; **p < 0.01. [file Image_2.tif]
